# Treatment experience of patients with failed *in vitro* fertilization-embryo transfer: a qualitative study from the perspective of the social ecosystem theory

**DOI:** 10.3389/fpubh.2026.1795539

**Published:** 2026-05-05

**Authors:** Yingkang Zhao, Keying Zhao, Meng Rao, Shuhua Zhao, Qingmei Liu, Zhenfang Su

**Affiliations:** 1 School of Nursing, Kunming Medical University, Kunming, Yunnan, China; 2 Reproduction and Genetics Department, The First Affiliated Hospital of Kunming Medical University, Kunming, Yunnan, China; 3 School of Public Health, Kunming Medical University, Kunming, Yunnan, China

**Keywords:** experience, failure of assisted conception, *in vitro* fertilization-embryo transfer, qualitative research, social ecosystems theory

## Abstract

**Background:**

A growing number of couples worldwide are seeking *in vitro* fertilization-embryo transfer (IVF-ET) to achieve pregnancy. However, failed IVF-ET treatment inflicts multidimensional stress on patients, involving individual, familial, and social levels, yet existing research has largely focused on single-dimensional analyses. Therefore, this study aims to systematically explore the treatment experiences of patients with failed IVF-ET using the Social-Ecological Systems Theory (SEST) as a guiding framework, in order to comprehensively understand the interplay of factors across individual, familial, and social levels.

**Methods:**

Based on the social-ecological systems theory, this study adopted a phenomenological qualitative research approach. From November 2024 to January 2025, patients who experienced failed *in vitro* fertilization-embryo transfer (IVF-ET) were recruited using purposive sampling for face-to-face, semi-structured interviews at a tertiary hospital in Kunming, Yunnan Province. Colaizzi's seven-step analysis method was applied to analyze the interview data.

**Results:**

A total of 15 patients with failed IVF-ET participated in this study, and three themes and eight sub-themes were derived from the interview data: (1) Microsystem: Experiencing physical and psychological distress (dynamic changes in complex emotional experiences; experiences of somatic discomfort); (2) Mesosystem: Confronting challenges of imbalance in economic status, family relationships and life patterns (excessive economic burden; barriers to maintaining family relationships; imbalance in life and work patterns); (3) Macrosystem: Weakness of the diversified external support system (insufficient informational support; need for improvement in medical service experience; inadequate policy support).

**Conclusion:**

Patients who experience failed IVF-ET-assisted pregnancy exhibit compromised social-ecological system status, accompanied by severe psychological distress and substantial economic burden, along with multiple adaptive challenges. These findings highlight the urgent clinical need to implement targeted psychological interventions, optimize medical support strategies, and mobilize multidisciplinary resources including medical staff, family, and social institutions, thereby improving the coping ability and long-term mental health outcomes of this population.

## 1 Introduction

Infertility is a globally recognized reproductive health disorder. The World Health Organization (WHO) defines it as follows: couples of reproductive age who have failed to achieve or maintain a clinical pregnancy after 12 months or more of regular unprotected sexual intercourse while living together (1). The prevalence of infertility is on a steady upward trend each year (2), and it has become the third most common health issue following cardiovascular diseases and tumors (3). The latest 2023 report released by the WHO indicates that approximately 17.5% of adults worldwide experience infertility at some point in their life course, with the prevalence rates standing at 17.8% and 16.5% in high-income countries and low- and middle-income countries, respectively (4). The prevalence of infertility in China has also risen markedly, increasing from 12 in 2007 to 18% in 2020, which is close to the average level in Asia (5).

Faced with the increasing prevalence of infertility, assisted reproductive technology (ART) has become an important clinical solution, among which *in vitro* fertilization-embryo transfer (IVF-ET) is the core modality (6). IVF-ET is a technology in which eggs retrieved from the female partner and sperm obtained from the male partner are fertilized and cultured *in vitro*; once the fertilized eggs develop into transferable embryos or blastocysts, they are then transferred into the female's uterine cavity (7). Since its first successful application in 1978, IVF-ET has revolutionized the treatment of infertility, bringing hope to millions of infertile couples worldwide (8). The number of individuals born via assisted reproductive technology (ART) has exceeded 6 million worldwide (9). This trend is also reflected in China, where the annual number of IVF-ET treatment cycles has exceeded 1 million, ranking among the highest in the world (10).

However, *in vitro* fertilization - embryo transfer (IVF-ET) is not only characterized by a complicated treatment process and high costs, but also many patients fail to achieve a successful transfer at one go (11). To be specific, according to the data on human assisted reproductive technology released by the Chinese Medical Association (CMA) in 2025, the current range of clinical pregnancy rates for fresh embryo transfer cycles in China is 39.65%−59.06%, with the live birth rate ranging from 28.69 to 50.16%; the pregnancy rate for frozen-thawed embryo transfer cycles is between 48.98 and 53.95%, and the corresponding live birth rate is 37.98%−43.05%(12). Therefore, the outcomes of pregnancy treatment with IVF-ET are highly uncertain, and patients undertaking each IVF-ET treatment cycle have to bear a certain degree of risk of failing to achieve clinical pregnancy (13). Studies have shown that IVF-ET treatment failure not only signifies the termination of physical therapy, but also triggers psychosocial crises, with patients commonly experiencing negative emotions such as anxiety, depression and self-blame (1). A comprehensive review by Neto et al. (14). Further highlights that such emotional challenges, alongside complex ethical and social considerations, are central to the psychosocial impact of assisted reproductive techniques, underscoring the need for holistic support and integrated mental health interventions. It can also result in the interplay between strained family relationships and social stigmatization, forming a multidimensional stressor (15, 16). Notably, the real experience of patients during the treatment process is closely related to their ability to cope with failure, subsequent recovery outcomes and treatment compliance (17). As a core dimension of medical service quality evaluation, patients' treatment experience plays a pivotal role in improving patients' recovery outcomes, enhancing treatment compliance, and increasing their satisfaction with medical visits (18). Therefore, it is imperative to explore the treatment experience of patients with failed IVF-ET treatment, as this exploration is crucial to addressing the aforementioned multidimensional stressors caused by treatment failure, improving patients' recovery outcomes, alleviating their physical and psychological discomfort, and assisting healthcare providers in formulating personalized intervention strategies.

However, existing research on the treatment experience of patients with failed IVF-ET treatment has mostly focused on single-dimensional analysis, neglecting the complex interactions between patients and their surrounding social environments (19). As noted earlier, failure of IVF-ET treatment not only triggers individual psychological crises but also leads to strained family relationships and social stigmatization, forming a multidimensional stressor that involves the individual, familial and social levels (20, 21). Therefore, there is an urgent need for a comprehensive and systematic theoretical framework to guide the in-depth exploration of these interrelated factors, and to fully grasp the connotation of patients' treatment experience and its influencing mechanisms. The Social-Ecological Systems Theory (SEST) provides a robust analytical tool for understanding the dynamic interactions between individuals and their social environments (22). This theory emphasizes that individual development is the outcome of continuous interactions between an individual and multi-layered environmental systems, and it categorizes the human social-ecological system into three types: the microsystem, mesosystem, and macrosystem (23). The microsystem refers to the individual system, encompassing biological, psychological, social and other subsystems that influence individual behavior; the mesosystem denotes small-scale groups exerting an impact on the individual system, including families, work groups and other social groups; the macrosystem represents systems and groups larger than small-scale ones, comprising organizations, institutions, communities and social cultures (24). Currently, this theory has been widely applied in research fields such as health promotion and health education, providing strong theoretical support for understanding individuals' health behaviors at different levels (25).

Therefore, drawing on the Social-Ecological Systems Theory, this study adopts a qualitative research method to comprehensively explore the treatment experience of patients with failed IVF-ET treatment through multi-level analysis, and to reveal the interactions between factors at different systemic levels and their comprehensive impacts on patients. To our knowledge, few studies have applied the Social-Ecological Systems Theory to explore the treatment experience of patients with failed IVF-ET, which fills the research gap of single-dimensional analysis and provides a new perspective for understanding the multidimensional stressors faced by these patients. Finally, the application of the Social-Ecological Systems Theory can lay a theoretical foundation for translating research findings into clinical practice, help healthcare providers and policymakers identify key intervention nodes in the individual, family, and social systems, and formulate targeted and holistic intervention strategies to improve patients' treatment experience and promote their physical and mental recovery.

## 2 Methods

### 2.1 Design

This study adopted a descriptive qualitative research method. This method emphasizes the role of life experience and is suitable for exploring individuals' lived experiences or events, obtaining interpretations from participants, thereby providing a new perspective on the essence of individual consciousness (26). This study aims to explore the treatment experience of patients who failed to achieve pregnancy through IVF-ET. Therefore, it is appropriate to adopt the method of descriptive phenomenology. This study followed the Consolidated Criteria for Reporting Qualitative Research (COREQ) (27).

### 2.2 Sample and setting

This study used purposive sampling. From October 2024 to January 2025, patients who had experienced failed to achieve pregnancy after IVF-ET were recruited from the Department of Reproductive Genetics of a tertiary hospital in Yunnan Province, China. The inclusion criteria were as follows: Female patients who were clearly diagnosed with infertility by specialists in the reproductive center; Patients who had experienced at least one failed embryo transfer; Patients who had normal ovarian function; Patients who were able to understand and express themselves in Chinese; Patients who voluntarily consented to participate in this study and signed a written informed consent form. The exclusion criteria were as follows: Patients with other severe mental disorders; Patients with other severe chronic diseases (e.g., malignant tumors). Patients experiencing major recent life stressors. The purpose of these exclusions was to protect the rights and wellbeing of the participants and ensure the quality of the data. The selection of research subjects followed the principle of maximum variation. The sample size was determined based on the saturation of data, i.e., when the data provided by the interviewees began to repeat and no new themes emerged from the analysis (28).

### 2.3 Data collection

An initial interview outline was designed and revised by four senior professionals with years of experience in qualitative research and reproductive medicine, after group discussions. The interview questions were pre-tested with two participants to verify the validity of the interview outline. Data from these two preliminary interviews were not included in the final results (29). The final interview outline was adjusted based on the interviewees' feedback and the effectiveness of the interviews. The detailed interview outline is provided in the Appendix1.

From November 2024 to January 2025, the researchers conducted face-to-face, semi-structured and in-depth interviews with the participants. A total of 15 female patients with infertility participated in this study. All face-to-face interviews were conducted by the first author (YKZ), who was a postgraduate student at a medical university, had received systematic training in qualitative research, and possessed experience in qualitative interviewing. All interviews were conducted in Chinese. No prior relationship had been established between the participants and the interviewers. After obtaining informed consent from each participant, the interview process was audio-recorded, and non-verbal information such as their facial expressions, tone of voice, and body movements was documented in a notebook. During non-treatment periods and patients' rest periods, we arranged a quiet place or an unused consulting room where only the interviewer and the participant were present, so as to ensure the smooth progress of the interview. Before the formal interviews, the interviewer introduced herself to the participants, explained the purpose, significance and methods of the interviews, and established a sense of trust with them. After obtaining consent, the interviewer scheduled an appropriate interview time with each participant. The duration of each interview ranged from 20 to 40 min. After the interviews started, the interviewer asked the participants about their general demographic information, including age, educational level, occupation, duration of infertility, and type of infertility. Then, the interview transitioned to the main interview outline. To ensure the completeness and authenticity of information, during the interview, the interviewer asked timely follow-up questions based on the participant's responses, but did not arbitrarily interrupt or guide the conversation. Meanwhile, after each participant completed the interview, we asked them if there was any additional information they needed to supplement.

Data collection was terminated when data saturation was achieved, and no new concepts were identified in the subsequent data analysis. Data collection continued until no new themes emerged from the interviews after conducting and analyzing one-on-one interviews with 13 participants, and the existing coding framework sufficiently captured the diverse experiences of the participants. To ensure that no new information would emerge, an additional two participants were interviewed. In total, 15 interviews were conducted, and data collection was terminated when saturation was confirmed.

### 2.4 Data analysis

The researcher (YKZ) transcribed the audio recordings verbatim within 24 h after the interviews were completed and organized the content into a formal document. Data analysis was conducted in Chinese, and then the results were translated into English and proofread by two independent bilingual translators, and the translated version was verified by other researchers to ensure that the original meaning was preserved across languages. Colaizzi's Seven-Step Analysis Method was adopted for data analysis (30). The specific steps were as follows: (1) Read the raw data transcribed from audio recordings carefully and repeatedly; (2) Identify and extract meaningful statements consistent with the research phenomenon; (3) Code the recurring meaningful viewpoints; (4) Summarize the coded viewpoints from the data; (5) Record the descriptions of each coded theme in detail and without omission; (6) Integrate, generalize, refine and derive themes from similar viewpoints; (7) Return the derived thematic concepts to the interviewees for verification. Two researchers (YKZ and ZFS) independently analyzed the interview data. When discrepancies arose, the two analysts first reviewed the original data to clarify their respective rationales, followed by a consensus meeting moderated by a third researcher. Any unresolved discrepancies were adjudicated by a senior researcher to reach a final decision. Finally, the comprehensive final results were constructed and confirmed through the review of all authors.

### 2.5 Rigor and trustworthiness

To ensure the trustworthiness of this qualitative study, the research adopted the framework of credibility, confirmability, transferability, and dependability proposed by Lincoln and Guba (31). To further address potential biases associated with the interviewer's status as a postgraduate student, multiple strategies were employed to ensure reflexivity and minimize interviewer bias. Credibility was enhanced through member checking, in which the preliminary findings were returned to several participants for verification and confirmation. In addition, peer debriefing was conducted with other experienced researchers to discuss and reflect on the data analysis process. To enhance transferability, the sample was selected to be as diverse as possible, and an in-depth, rich description of the participants' demographic characteristics and study results was provided. Dependability was ensured by providing a detailed description of the research methods and involving peers in the analytical process. To ensure confirmability, the principal researcher maintained a reflective journal to document personal assumptions and potential biases, which was regularly discussed with the research team. In addition, independent researchers cross-checked the data and coding process to minimize subjective bias.

### 2.6 Ethics

This study has been approved by the Ethics Committee of the First Affiliated Hospital of Kunming Medical University (reference number: 2024-L-284). All participants signed a written informed consent form, agreeing to the audio recording and documentation of the interviews. The participants were assured that they had the right to withdraw from the study at any time without providing any reason, and that their non-participation or withdrawal would incur no consequences. They could decline to answer any specific questions. No compensation was provided for participation. The content of the interviews was kept anonymous and confidential and was used solely for the purpose of this study. No identifiers of individual participants or any images were included were included in the manuscript or Supplementary material.

## 3 Results

### 3.1 Participant characteristics

A total of 15 patients who had experienced IVF-ET treatment failure were interviewed in this study. The age of the interviewees ranged from 25 to 45 years, with a mean age of (34.80 ± 6.29) years. Among them, 1 patient (6.7%) had a duration of infertility exceeding 10 years and 4 patients (26.7%) had a duration of infertility exceeding 5 years. Additionally, 7 cases (46.7%) were diagnosed with secondary infertility and 8 cases (53.3%) with primary infertility see Table 1.

**Table 1:** General information of the interviewees (*n* = 15).

ID	age	Ethnicity	Residential region	Educational level	Occupation	Avg. monthly HH income (CNY)	Duration of infertility	Type of infertility	Months since last IVF-ET failure
N1	40	Han	Rural	Primary school	Farmer	3,001~4,000	4	Secondary infertility	3
N2	25	Han	Urban	Junior high school	Unemployed	2,001~3,000	3	Primary infertility	6
N3	43	Han	Urban	Primary school	Unemployed	2,001~3,000	3	Secondary infertility	4
N4	40	Han	Urban	College	Staff member	4,001~5,000	4	Primary infertility	3
N5	38	Yi	Rural	Senior high school	Self-employed	4,001~5,000	2	Secondary infertility	9
N6	30	Han	Urban	College	Staff member	5,001~6,000	6	Primary infertility	4
N7	34	Bai	Rural	Junior high school	Self-employed	3,001~4,000	13	Primary infertility	7
N8	28	Han	Urban	College	Staff member	5,001~6,000	1	Primary Infertility	10
N9	26	Han	Urban	Junior high school	Freelancer	4,001~5,000	4	Primary infertility	5
N10	34	Yi	Rural	Senior high school	Self-employed	3,001~4,000	2	Secondary infertility	3
N11	26	Han	Rural	Junior high school	Unemployed	3,001~4,000	3	Primary infertility	2
N12	35	Han	Urban	College	Staff Member	4,001~5,000	6	Primary infertility	3
N13	40	Han	Urban	Senior high school	Self-employed	4,001~5,000	4	Secondary Infertility	3
N14	45	Han	Rural	College	Unemployed	3,001~4,000	2	Secondary Infertility	6
N15	38	Miao	Urban	College	Civil servant	5,001~6,000	8	Secondary Infertility	5

### 3.2 Main findings

**Figure 1:**
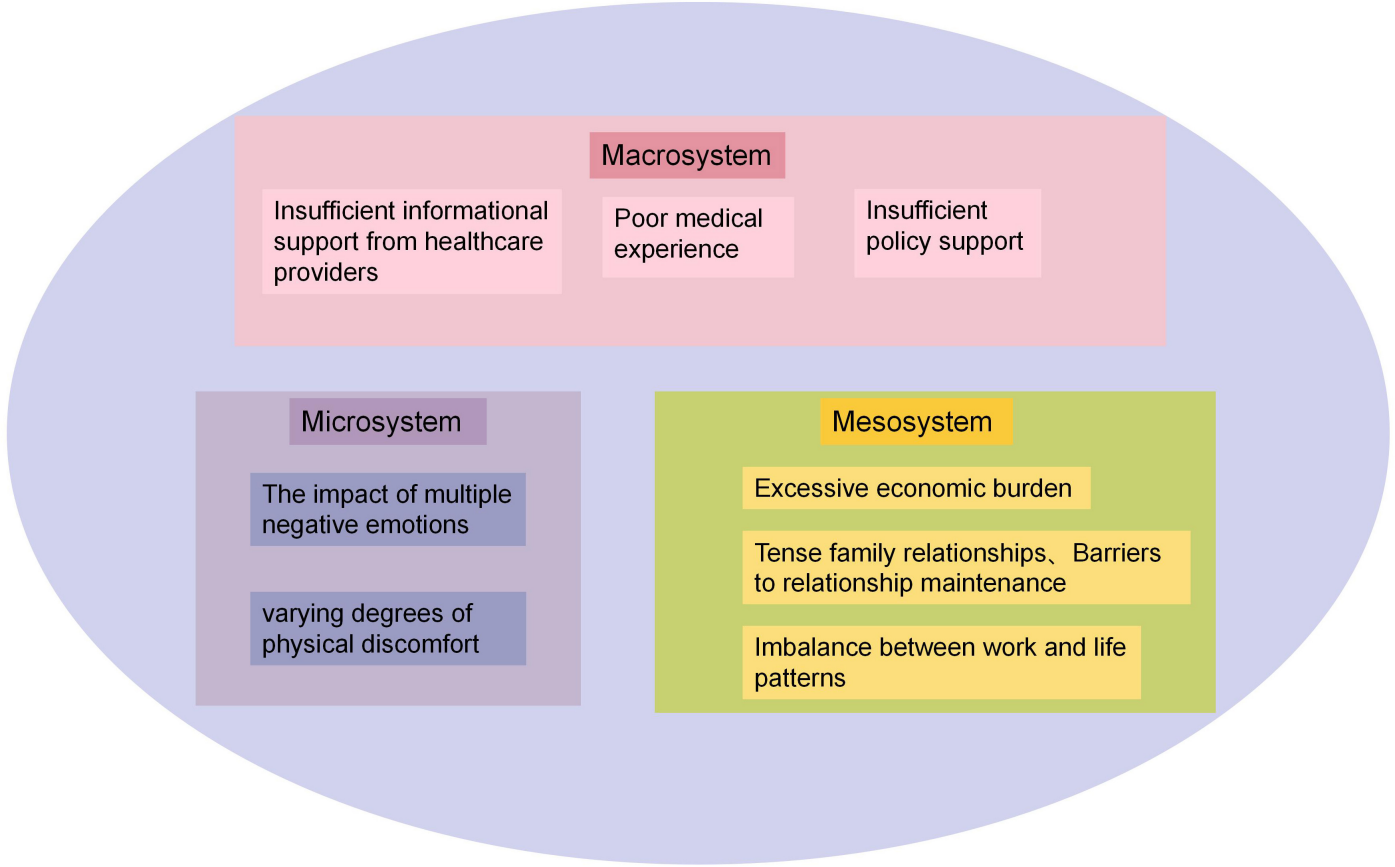
Theme and subthemes.

Based on the Social-Ecological Systems Theory and Colaizzi's Seven-Step Analysis Method, the treatment experiences of patients who had failed IVF-ET assisted reproduction were categorized into three main themes and eight subthemes see Figure 1.

**Theme 1: Microsystem: Experiencing Physical and Psychological Distress**

**Subtheme 1: The experience of dynamic changes in complex emotions**

The majority of patients with infertility, during their first medical visit, believe that they will eventually achieve a successful pregnancy as long as they undergo IVF-ET treatment, and they are fully confident in the treatment.

“*After the embryo transfer at the beginning, I still had quite high expectations. Since I had a good number of embryos and everything had been going smoothly for me along the way, I thought it might work out on the first try” (N11)*.

Patients usually hold great expectations for the outcome of the embryo transfer after the procedure. When the embryo transfer results in failure, patients often experience negative emotions such as sorrow, grief, and self-blame.

“*Unfortunately, the embryo failed to implant after the transfer, and I felt suddenly lost and sad” (N11). “After the failed embryo transfer, we have been under quite a lot of psychological pressure” (N4)*.

Some patients tend to look for the causes within themselves, believing that certain behaviors of theirs led to the failure of the embryo transfer, and thus get trapped in a spiral of self-blame.

“*I can't help but wonder if there was something I overlooked that led to the failed embryo transfer. I've been obsessing over these thoughts all day long, feeling unmotivated to do anything and overcome with anxiety” (N12)*.

When patients decide to undergo another embryo transfer, the experience of the previous failed transfer imposes psychological stress on them, often leaving them feeling anxious, tense and fearful.

“*Now that I'm preparing for another transfer, I can't help thinking—what if it fails again like last time? The psychological pressure is quite heavy, and I feel a lot of worry and fear” (N7)*.

**Subtheme 2: Experience of physical discomfort**

Patients undergoing IVF-ET for assisted pregnancy will go through several treatment stages, including controlled ovarian hyperstimulation, oocyte retrieval, embryo transfer, and luteal phase support. Patients endure varying degrees of physical suffering due to numerous invasive procedures and medication side effects.

“*I have to inject myself in the abdomen every single day, and my stomach doesn't feel as comfortable as it used to. On top of that, I have to insert progesterone suppositories daily, which makes the vaginal area really sore and uncomfortable too” (N5)*.

The physical toll was not limited to the treatment itself; it also manifested in bodily changes that patients found distressing.

“*I used to weigh only 46.5 kg, but after starting IVF treatment, I've gained weight to 60 kg now” (N1)*.

For some patients who have experienced IVF-ET failure may have already gone through 1~2 treatment cycles, and the prolonged course of treatment has left them feeling extremely exhausted. The physical demands of repeated procedures left visible and tangible marks.

“*I had to get an injection every single day during the controlled ovarian hyperstimulation. Now my abdomen is covered with injection marks, and sometimes after the injections, there are a lot of hard lumps on my stomach—they hurt unbearably even when I accidentally bump into them” (N2)*.

Beyond the clinical procedures, the logistical burden of treatment further intensified physical and emotional fatigue.

“*I have been undergoing nonstop examinations since the start of the year, having to go to the hospital almost every single day. What's more, I don't live in Kunming, so the daily back-and-forth commuting has left me utterly exhausted” (N3)*.

**Theme 2: Mesosystem: Facing Challenges of Economic Burden, Family Relationship Tensions and Life Pattern Imbalance**

**Subtheme 1: Excessively heavy economic burden**

The cost of assisted pregnancy treatment via IVF-ET is relatively high, and many patients feel burdened by the significant financial pressure brought by the treatment.

“*Each injection costs me over 1,300 yuan. How many people can actually afford this” (N10)*.

For patients who have experienced embryo transfer failure, the economic burden is even more pronounced compared to those undergoing their first transfer. These patients often face repeated cycles, each carrying its own substantial costs.

“*The quality of the embryos formed after each egg retrieval has never been satisfactory, but the cost of undergoing third—generation IVF would still be quite high for me” (N1)*.

The uncertainty of treatment success compounds the financial pressure, creating a sense of precarious investment.

“*Now I'm about to start a new embryo transfer cycle, which will cost a fortune again. I have no idea if it will actually work this time. If it fails again, wouldn't all that money go down the drain? Sometimes I feel like this is just a bottomless pit” (N12)*.

**Subtheme 2: Barriers to maintaining family relationships**

After failing to achieve pregnancy through IVF-ET, the long—unfulfilled desire for pregnancy can undermine the couple's fertility goals and become a potential crisis in their marital relationship.

“*My husband and I both feel that our family might go the distance only if we have a child together. If we never have a baby, I'm afraid we can never be certain about our future as a couple” (N1)*.

Moreover, the prolonged course of treatment and the side effects of medications have led to a decline in sexual life satisfaction, which in turn has affected the relationship between couples.

“*During the treatment, I have to use vaginal suppositories quite often, which has also taken a toll on my sex life—I just don't feel interested in it anymore” (N10)*.

At the same time, rooted in traditional Chinese views on childbearing, the inability to conceive is frequently attributed to women, placing them under heightened scrutiny from male partners or extended family members.

“*We live in a rural area. My parents-in-law firmly believe that we must have a child of our own. What's more, I'm certain they still think I'm too old for that” (N3). “No one else in the family knows that we're doing IVF—only my husband and I know about it. Because where we live, in a rural area, people think that being unable to have a baby is something shameful” (N9)*.

**Subtheme 3: Imbalance between life and work patterns**

Patients undergoing IVF-ET treatment often have to make frequent trips to the hospital for examinations and therapies. As a result, some of them choose to quit their jobs, making infertility treatment the sole focus of their lives.

“*I'm not working anymore now; I'm just staying at home waiting for the embryo transfer” (N2)*.

Patients who choose to continue working often face increased occupational stress due to conflicts between work commitments and infertility treatment.

“*During the ovulation induction cycle, I have to come back frequently for ovulation monitoring, which means I need to take time off work quite often. Sometimes, the monitoring takes a really long time, and when I get back to work, there's still a ton of tasks waiting for me. In the end, I have to use my own rest time to catch up on the work” (N11)*.

**Theme 3: Macro-level System: Insufficient Diversified External Support Systems**

**Subtheme 1: Insufficient information support**

Virtually all respondents indicated that they lack knowledge related to reproductive health, find it difficult to distinguish the authenticity of information available online.

“*This is my first time going through this process, but there are so many things I don't know. I have nowhere to turn to for answers, and I'm not sure if the information I find online is accurate” (N9)*.

Despite regular clinical encounters, many felt that their informational needs were not adequately addressed. This gap was particularly pronounced regarding practical aspects of self-care following treatment failure.

“*After my last embryo transfer failed, many people told me I should pay attention to my diet and adjust my daily routine for better conditioning, but I really have no idea how to do these things specifically” (N5)*.

**Subtheme 2: The medical service experience needs to be improved**

Due to the limitations of medical standards, only a small number of municipal or provincial Grade A tertiary hospitals have established reproductive genetics centers. For patients living far away from these hospitals, frequent travel between home and the hospital leads to increased physical exhaustion, time costs and financial expenses.

“*I wonder if some of these procedures could be done without having to come to the hospital in person. In my opinion, a phone call would suffice for communication. It's really inconvenient for people like us who live far away and have to take time off work for these frequent back-and-forth trips to the hospital” (N6)*.

The scarcity of specialized resources also translates into prolonged waiting times, adding to the daily strain of treatment. Even routine procedures become time-consuming ordeals.

“*It's already quite challenging to get an injection every day. Sometimes, there are long queues for injections, forcing me to wait for ages, and each time it takes up a lot of my time” (N8)*.

Additionally, healthcare providers pay insufficient attention to patients' emotional wellbeing, leaving some patients feeling that the treatment process is nothing more than an impersonal assembly line.

“*I hope doctors can give me specific guidance on what the next treatment plan should be, instead of treating me like an assembly line process every time” (N10)*.

**Subtheme 3: Insufficient policy support**

The medical costs required for IVF-ET assisted reproduction are relatively high, and patients have a strong demand for medical security. Although the costs of *in vitro* fertilization and embryo transfer treatment have been included in the scope of medical insurance at present, the reimbursement rate remains low due to certain policy restrictions, leaving them to shoulder a substantial portion of expenses.

“*Even though IVF treatments are now covered by medical insurance, the costs of laboratory tests, medical examinations and medications are still fully self-funded” (N10)*.

For patients from outside the treatment region, the financial burden is further compounded by the non-portability of insurance benefits.

“*I am from another province, so my medical insurance is not applicable here. If the treatment doesn't work out, the over 20,000 yuan spent will be totally wasted. The cost is just too high, and both the time and financial costs are quite substantial” (N8)*.

## 4 Discussion

### 4.1 Multi-level ecosystem imbalance and cultural influences on patients' experiences

Patients who have failed to achieve successful pregnancy via IVF-ET are burdened with excessive life pressure and heavy psychological stress due to various stressors (32), which in turn leads to the imbalance of their social ecosystem. From the interview results, at the micro-system level, failed assisted reproduction directly exacerbates patients' negative psychological experiences, such as anxiety, depression and loss of self-worth. Meanwhile, due to the particularity of the treatment, patients also suffer varying degrees of physical distress. At the meso-system level, the main manifestations lie in family and workplace scenarios. The couple's expectations for childbearing, as well as the excessive concern or inappropriate remarks from relatives and friends, constitute invisible sources of social pressure. Meanwhile, the need for frequent hospital visits takes a toll on work performance and career development. At the macro-system level, the insufficient coverage of policies and medical security related to assisted reproductive technology fails to meet the patients' needs. It is evident from the above that the predicament faced by patients who have experienced IVF-ET failure is a comprehensive issue embedded in a multi-level ecosystem. The various systems interact with one another. The psychological crisis in the micro-system may be exacerbated by the absence of support from the meso-system, while the cultural norms and policy environment of the macro-system define the boundaries for the entire response process (33). This multi-level interplay aligns with the findings of Neto et al. (14), who emphasize that addressing the psychosocial challenges of ART requires holistic support, integrated mental health interventions, and culturally sensitive counseling approaches that consider the full social-ecological context of patients' lives.

The experiences and perceptions of Chinese women following IVF-ET failure are deeply shaped by traditional Chinese cultural norms. Central to these is the family-oriented culture and strong emphasis on filial piety, which positions childbearing as a core social and moral obligation for women. In Chinese society, infertility is often stigmatized and viewed as a failure to continue the family line, leading to shame, guilt, and pressure from family and the wider community (34). Women tend to internalize blame and suppress negative emotions to maintain family harmony, reflecting cultural values of collectivism and emotional restraint. In contrast, many Western cultures emphasize individual autonomy, personal wellbeing, and psychological openness. Infertility is more often regarded as a medical condition rather than a moral shortcoming, with less social stigma and greater willingness to seek psychological support (35). These cultural differences result in distinct coping patterns: Chinese women tend to adopt inward, passive coping, whereas Western women are more likely to express emotions and seek help actively. Therefore, cultural background is essential for understanding the unique distress, coping styles, and care needs of Chinese patients with IVF-ET failure.

### 4.2 Micro-level: IT is necessary to implement personalized nursing interventions to promote the physical and mental comfort of patients

Failure to achieve a successful pregnancy via IVF-ET not only dashes the patients' short-term hopes of conceiving, but also subjects them to physical and emotional distress (36). In this study, we found that at the psychological level, the patients' mental states are continuously and dynamically changing, yet they often endure immense emotional distress, with negative emotions such as anxiety, depression, sorrow, and self-blame likely to emerge (37). This is consistent with the research findings of Kumar (38). During the long-term infertility treatment process, patients are filled with great expectations for a successful pregnancy. However, they tend to experience intense feelings of loss after treatment failure, and may even begin to doubt their own self-worth; some patients may further develop a crisis of self-identity (39). Simultaneously, the psychological stress imposed by the patient's spouse and family members can further aggravate her psychological burden, including their reproductive expectations and the traditional societal constructs of the maternal role, thereby rendering the patient trapped in a psychological dilemma (40). At the physiological level, undergoing procedures such as ovarian stimulation, oocyte retrieval, and embryo transfer may lead to complications like ovarian hyperstimulation syndrome (OHSS) in patients, which manifests as uncomfortable symptoms including abdominal distension, abdominal pain, and nausea (41). Multiple invasive procedures also exacerbate the experience of physical distress, which in turn further aggravates negative psychological feelings, forming a self-reinforcing vicious cycle of mind-body interaction (21). For psychological distress, healthcare providers should conduct routine, structured psychological assessments and provide timely emotional support and professional counseling. They can guide patients to reappraise negative experiences, focus on adaptive coping, reflect on the personal meaning of fertility, and cultivate positive adjustment strategies through psychological counseling (42), narrative nursing (43), and cognitive behavioral therapy (44), which are crucial to facilitating post-traumatic growth and emotional recovery. Furthermore, individualized psychological follow-up plans should be formulated according to the duration of distress and intensity of negative emotions. Peer support groups can also be established to allow patients to share experiences, obtain emotional resonance, and reduce feelings of isolation (45). For physical discomfort, the clinic should implement a dedicated primary nursing responsibility system. With assigned nurses providing continuous, whole-course follow-up and developing symptom-specific and stage-based nursing plans (46). Personalized care not only focuses on the relief of immediate symptoms, but also the restoration of self-identity, autonomy, and sense of control (47), enabling them to achieve physical and mental comfort in the dual dimensions of physical wellbeing improvement and psychological resilience enhancement.

### 4.3 Mesosystem: it is necessary to establish a sound and stable social support system to enhance patients' sense of being supported

Influenced by traditional Chinese fertility concepts and confronted with the frustration of unmet couple fertility goals, family support exhibits dual characteristics of emotional alienation and functional weakening. The failure of assisted reproduction may trigger emotional conflicts, mutual accusations, and even marital crises between couples, which is consistent with the findings of Kim's research (48). A number of patients also face fertility-related pressure and a lack of understanding from their parents' generation, which results in the impairment of the family's function as a core source of support (49). The majority of patients in this study have to take frequent hospital visits for treatment, which forces them to resign from their jobs or adversely affects their work performance. This leads to an imbalance in their life and work patterns, a certain degree of impact on their financial resources, an increase in illness-related stigma, and ultimately results in the breakdown of their occupational support network (50). Previous studies have found that family and social interactions form the foundation of a patient's social support network, with the support from family members and friends serving as its core pillar. Patients who receive a high level of emotional support tend to demonstrate greater enthusiasm for treatment (51). To address these challenges, a meso-level support system requires collaborative and actionable strategies across family, workplace, and clinical settings. First, at the family level, clinicians should actively involve family members in supportive interventions, guide them to establish rational fertility attitudes, and conduct family-centered health education to improve communication and reduce blame. Families should be encouraged to provide consistent listening, companionship, and practical assistance, strengthening emotional support functions and improving patients' perceived support (52). Second, at the workplace level, governments and enterprises should jointly optimize labor protection policies for individuals undergoing infertility treatment. Concrete measures include flexible working hours, protected medical leave, workload adjustment, and anti-discrimination regulations. In addition, workplace training on fertility stigma and humanistic care should be strengthened to create an inclusive, supportive environment rather than merely advocating abstract tolerance.

### 4.4 Macro-level system: improve the external support system and medical service system to meet the diverse needs of patients

Although the number of medical institutions approved to provide assisted reproductive technology (ART) in China has been on the rise in recent years, these facilities remain heavily concentrated in economically developed regions. The lack of assisted reproductive medical resources in primary medical and health institutions, which forces patients to make frequent trips to designated medical institutions, along with the shortage of high-level medical and health resources, has to a certain extent become a barrier to the patient's medical experience (53). Patients are unable to obtain professional information support from the community, and the lack or ambiguity of information is a key factor exacerbating psychological distress following IVF-ET failure (54). At the same time, this study found that patients who have experienced IVF-ET failure face high and ongoing financial burdens. Although assisted reproductive treatment has now been included in the scope of medical insurance, due to policy restrictions, the reimbursable ratio remains relatively low. As a result, financial burden continues to be the primary source of stress for patients who have experienced *in vitro* fertilization-embryo transfer failure (55). To resolve these gaps, a multilevel macro-support system must be established. First, primary healthcare systems should be upgraded to include basic reproductive health services and post-failure follow-up care. Reproductive health knowledge should be popularized through community education. Standardized, stage-specific information manuals and official mini-programs should be developed for patients with IVF-ET failure, ensuring accurate, consistent, and trustworthy information delivery. Second, high-level hospitals should optimize service models by establishing one-stop reproductive medicine centers, expanding telemedicine, and improving appointment and follow-up systems to reduce travel burden and time costs (56). Clinicians should replace rigid, formulaic communication with individualized, empathetic interaction to enhance patient-centered care and medical experience (57). Finally, the government should further expand and refine insurance policy for assisted reproduction, increasing reimbursement ratios, extending coverage to repeated cycles and post-failure management, or providing special financial subsidies for low-income patients. Such policy improvements are essential to reduce long-term economic stress and improve equity in access to care.

## 5 Limitations

Several limitations of this study should be acknowledged. First, data were collected solely through face-to-face semi-structured interviews, without the use of participant or non-participant observation. While interviews are appropriate for exploring patients' subjective experiences, the absence of observational data may limit the richness of contextual information, such as non-verbal behaviors and real-time interactions between patients and their social environment, which could have further enriched the findings. Second, only patients in the pre-embryo transfer period were interviewed in this study, which means the findings may not fully capture the long-term experiences of patients with IVF-ET treatment failure across the entire treatment continuum, from initial diagnosis to successful pregnancy or definitive outcomes. Third, all participants were recruited from a single hospital in one region of China. This single-center, regionally focused design inherently limits the generalizability of the findings, as the results may reflect institution-specific practices and regional characteristics that are not representative of other settings. Fourth, this study was conducted within a Chinese cultural context. Different cultural, social, or healthcare system environments may give rise to distinct experiences and perspectives, further constraining the transferability of our conclusions to other populations or countries. To address these limitations, future multicenter studies involving diverse geographic regions and healthcare settings, as well as cross-cultural comparative research, are needed to validate and extend the generalizability of the current findings. Finally, this study explored various problems and demands faced by patients solely from the patients' perspective, and lacked attention to the perspectives of other relevant stakeholders, such as medical staff, patients' family members, medical institutions, and relevant policy-making departments. The absence of an investigation from a multi-stakeholder perspective may result in the countermeasures proposed by the study failing to fully balance the needs of all parties, thereby affecting the comprehensiveness and feasibility of these countermeasures. To address this limitation, future research should incorporate the perspectives of multiple stakeholder groups. Such multi-stakeholder approaches would enable the development of more comprehensive, balanced, and implementable interventions that align with the needs and capacities of all parties involved in IVF-ET care.

## 6 Conclusion

Based on the social—ecological systems theory, this study explored the treatment experiences and needs of patients who experienced *in vitro* fertilization-embryo transfer failure from three microsystem levels, namely the micro, meso, and macro systems. This study reveals that patients who failed to achieve pregnancy through IVF-ET are confronted with an impaired social ecosystem. The interconnections and interactive influences among various systems exacerbate patients' psychological and financial burdens, thereby exposing them to additional pressures and challenges in coping with infertility. In the future, it is necessary to raise awareness of patients with infertility who have experienced IVF-ET failure and integrate multi-stakeholder resources to maintain the stability of their social ecosystem.

## Data Availability

The raw data supporting the conclusions of this article will be made available by the authors, without undue reservation.
