# Supplementary material for: Treatment experience of patients with failed in vitro fertilization-embryo transfer: a qualitative study from the perspective of the social ecosystem theory
Source: Front Public Health. 2026 May 5;14:1795539. doi: 10.3389/fpubh.2026.1795539 (PMC13183816; doi:10.3389/fpubh.2026.1795539)
Supplement: Supplementary file 1 [file Supplementary_file_1.docx]

Supplementary Material: Semi-Structured Interview Guide

| **Introduction**   - Before starting the interview, the researcher introduced themselves, explained the purpose of the study, assured confidentiality, and explained that the interview would be audio-recorded. - Written/oral informed consent was obtained from all participants. | | |
| --- | --- | --- |
| **S/N** | **Main question** | **Probes (if necessary)** |
| 1 | Could you share some of your experiences with seeking infertility treatment? | - Can you tell me about your first consultation? - What was the most difficult part of the treatment process for you? |
| 2 | What are your feelings about undergoing IVF-assisted pregnancy again? | - How do you feel this time compares to your previous attempts? - What are your hopes or concerns? |
| 3 | What psychological changes have you experienced after the embryo transfer failure? Could you please describe them in detail? | - Can you describe a typical day after learning the results? - How did you cope with these feelings? |
| 4 | What attitudes have your family and friends held towards your embryo transfer failure? | - Are there any specific conversations or moments that stood out to you? - Whose support has been most meaningful to you? |
| 5 | What do you think have been the biggest impacts and changes on your family, life and work since the embryo transfer failure? | - Has this affected your daily routines? - Has it impacted your relationship with your partner? |
| 6 | What kind of care and support would you expect from your family members, doctors and nurses? | - What would ideal support from medical staff look like to you? - What do you wish your family understood better? |
| 7 | What aspects of support and assistance do you need from your family and society? | - Besides family, what kind of social resources (e.g., counseling, peer groups) do you think would be helpful? |
| **Closing**  After completing the core questions, the researcher asked: “Is there anything else you would like to share about your experience that we haven’t covered today?” Participants were thanked for their time and offered the opportunity to ask questions about the study. | | |
